# Alternative Splicing in *Trichophyton rubrum* Occurs in Efflux Pump Transcripts in Response to Antifungal Drugs

**DOI:** 10.3390/jof8080878

**Published:** 2022-08-20

**Authors:** Marcos E. R. Lopes, Tamires A. Bitencourt, Pablo R. Sanches, Maíra P. Martins, Vanderci M. Oliveira, Antonio Rossi, Nilce M. Martinez-Rossi

**Affiliations:** Department of Genetics, Ribeirão Preto Medical School, University of São Paulo, USP, Ribeirao Preto 14049-900, SP, Brazil

**Keywords:** *Trichophyton rubrum*, multidrug resistance, alternative splicing, dermatophytoses, antifungal therapy, biofilm

## Abstract

Dermatophytes are challenging to treat because they have developed many strategies to neutralize the stress triggered by antifungals. Drug tolerance is achieved by mechanisms such as drug efflux and biofilm formation, and cellular efflux is a consequence of the synergistic and compensatory regulation of efflux pumps. Alternative splicing (AS) has also been considered as a mechanism that enhances fungal adaptive responses. We used RNA-seq data from the dermatophyte *Trichophyton rubrum* exposed to undecanoic acid (UDA) to search for and validate AS in genes encoding efflux pumps. The magnitude of this phenomenon was evaluated using UDA and other antifungals (caspofungin, itraconazole, and terbinafine) in planktonic and biofilm cultures. In addition to the conventional isoforms, the efflux pump encoded by TERG_04309 presented two intron-retained isoforms. Biofilms trigger the simultaneous production of at least two isoforms. The intron-retained isoforms showed short lengths and topologically different organization. Furthermore, we identified the putative interaction of efflux pumps (TERG_04309 and TERG_04224). Co-expression of these genes suggests a synergistic action in antifungal resistance. Our data provide new insights into drug tolerance related to differential isoform usage and the co-expression of stress-responsive genes, which may lead to higher antifungal resistance, mainly in biofilms.

## 1. Introduction

*Trichophyton rubrum* is the main causative dermatophyte of superficial mycosis worldwide [1]. This species infects the corneum stratum of the epidermis and nails but is rarely found in cases of hair infections [1]. It is estimated that 25% of the world’s population suffers from dermatophytosis [2]. The clinical treatment of dermatophytosis is challenging, mostly because of the limited antifungal arsenal which is restricted to a few molecular targets [3,4]. Moreover, the antifungal-resistance phenotype is an issue in this complex scenario. This molecular mechanism confers resistance to antifungal drugs and restricts treatment options [5].

Dermatophytes activate different mechanisms to counteract the toxic effects of antifungal compounds. Some strategies to overcome antifungal toxicity are drug degradation, biofilm formation, mutations in targeted genes, overexpression of genes that encode chaperones, and the upregulation of multidrug efflux transporter genes, including ABC-type transporters [2]. These are transmembrane proteins that extrude chemically unrelated compounds, decreasing their concentration within fungal cells [6]. The enrichment of these genes in the dermatophyte genomes [7], the occurrence of drug-specific activation of efflux pumps [8,9], and their compensatory activities in response to antifungal stress [3,10] strengthen the relevance of the efflux mechanism as an adaptive strategy used to assure dermatophyte survival.

Moreover, biofilm confers more antifungal tolerance than planktonic cells [11,12]. Biofilms are a complex of cell populations associated with surfaces that are incorporated into the extracellular matrix. They are responsible for a broad spectrum of infections in humans [4]. Biofilm formation depends on active cell communication and regulatory mechanisms [13,14], which contribute to its adaptation to antifungals. Furthermore, extracellular matrix and metabolic heterogeneity are factors related to antifungal resistance in biofilms [4].

One strategy to overcome antifungal resistance is to promote alternative splicing (AS), as previously reported for *T. rubrum* [15,16,17,18]. The predicted occurrence of AS in genes that encode efflux pumps in *T. rubrum* in response to the antifungal agents undecanoic acid (UDA) [15] and acriflavine (ACR) [3] is indicative of the association between splicing changes and drug resistance. AS is a regulatory process at the post-transcriptional level that leads to the production of different mRNAs from a single gene. A deep understanding of this mechanism and its consequences for the fungus are yet to be achieved [3,15].

Efforts to expand our knowledge about the molecular events associated with antifungal resistance are fundamental for improving antifungal therapy. In this context, this study aimed to analyze the occurrence of AS in efflux pump-encoding genes under different drug exposure conditions in both planktonic and biofilm cells of *T. rubrum* to pave new ways to understand the adaptive mechanisms tuned by antifungal stimuli.

## 2. Materials and Methods

### 2.1. Strains and Culture Conditions

The *T. rubrum* strain CBS 118892 was obtained from the Westerdijk Fungal Biodiversity Institute (formerly CBS-KNAW, Utrecht, Netherlands). This strain was cultivated in malt extract agar (MEA) medium (2% glucose, 2% malt extract, 0.1% peptone, and 2% agar, pH 5.7) at 28 °C for 21 days. The conidia suspension was prepared by flooding the plates with 0.9% sterile NaCl solution and filtering through fiberglass to remove hyphal fragments. The conidial concentration was then estimated using a Neubauer chamber.

Approximately 1 × 10^7^ conidia were inoculated in 50 mL of RPMI medium (Gibco) and incubated at 28 °C for 96 h under constant agitation (120 rpm). The resultant mycelia were transferred to 100 mL of fresh RPMI medium containing sub-inhibitory concentration (70% minimum inhibitory concentration — MIC) of the four following antifungals: undecanoic acid (UDA, 17.5 μg/mL) [15], caspofungin (CASP, 56 μg/mL) (unpublish result), itraconazole (ITR, 0.56 μg/mL) [19], and terbinafine (TRB, 0.14 μg/mL) [19], for 1 and 12 h. *T. rubrum* was also cultivated in RPMI medium without the drugs as a negative control. The cultures were filtered after each time point, and the mycelia were used for RNA extraction. The experiments were performed in triplicates.

### 2.2. Drug Exposure in a Biofilm Environment

The biofilm formation assay was performed as previously described [20] with some modifications. *T. rubrum* was cultivated in MEA medium at 28 °C for 10 days, and the conidial suspensions were prepared and adjusted to a final concentration of 1 × 10^6^ conidia/mL. This suspension was added to 24-well plates and incubated for 4 h at 37 °C for pre-adhesion. Then, the supernatant was gently removed from the wells, followed by the addition of 1 mL of RPMI medium (containing 2% glucose, buffered with 3-(N-morpholino) propane sulfonic acid (MOPS)). The plates were incubated at 37 °C for 96 h, at which the maturation stage of biofilms was achieved, as previously shown [21]. After that, fresh RPMI medium and antifungal agents were added under the following conditions: control (RPMI medium), TRB in 70% and 100% of MIC (0.14 µg/mL and 0.20 µg/mL of TRB, respectively), and CASP in 70% and 100% of MIC (56 µg/mL and 80 µg/mL CASP, respectively). All these conditions were maintained at 37 °C for an additional 1 h. All experiments were performed in triplicates. To prepare these samples for RNA extraction, the biofilm was washed with 1 mL of saline solution, removed from each well by pipetting, and centrifuged at 1.377× *g* for 10 min. The resultant pellet was resuspended in 500 µL of lytic solution (20 mg of lysing enzymes, (Sigma-Aldrich, St Louis MO, USA) 3 M KCl, pH 6.8), followed by incubation for 1 h at 28 °C with agitation (120 rpm), as previously described [22].

### 2.3. RNA Extraction and Cdna Synthesis

Total RNA extraction was performed using the Illustra Spin RNA Isolation Kit (GE Healthcare, Chicago IL, USA). According to the manufacturer’s instructions, the RNA samples were treated with DNAse I (Sigma-Aldrich, St Louis MO, USA) and converted to cDNA using a High Capacity cDNA Reverse Transcription Kit (Applied Biosystems, Waltham MA, USA). To assess the quality of the cDNAs obtained, PCR was performed using oligonucleotides to amplify a region of a constitutive gene that codes for β-tubulin.

### 2.4. In Silico Analyses to Characterize the ABC Transporter Isoforms

We selected intron retention events involving efflux pump-encoding genes from the RNA-Seq data [3,15]. The in silico prediction of the resultant proteins from conventional and alternative splicing was conducted using sequences retrieved from the Ensembl fungi database (https://fungi.ensembl.org/index.html, accessed on 8 January 2019). Reading frames and domains were predicted using the Expasy translate tool (https://web.expasy.org/translate/, accessed on 8 January 2019) and the InterPro database (https://www.ebi.ac.uk/interpro/, accessed on 10 January 2019), respectively.

The alignment of the efflux pump protein encoded by the TERG_04309 gene was carried out against different dermatophytes and non-dermatophyte fungi using the blast package in Ensembl fungi (https://fungi.ensembl.org/Multi/Tools/Blast, accessed on 4 February 2021). We performed graphical representations of isoforms and resultant proteins using the Illustrator of biological sequence (IBS) 1.0 software (CUCKOO Workgroup, Hongshan District, Wuhan, Hubei Province, China) [23].

### 2.5. In Silico Analysis of Genic Interaction Network

To predict the genes that can establish a genic network with TERG_04309, this gene was used as input on the STRING platform (https://string-db.org/, accessed on 4 October 2019) with a minimum required interaction score of 0.150 and a maximum of 10 interactions.

### 2.6. Alternative Splicing Validation—Gene Expression Analysis

The construction and quality analysis of the primers used in RT-qPCR assays were performed using the DNA primer quest tool (http://www.idtdna.com/primerquest/Home/Index — accessed on 12 January 2022) and oligoanalyzer 3.1 (https://www.idtdna.com/calc/analyzer, accessed on 12 January 2022) from IDT, and the BLAST tool (http://blast.ncbi.nlm.nih.gov/Blast.cgi, accessed on 12 January 2022). The primers used in these assays are listed in Table S1.

Oligonucleotides were designed within the region of the analyzed intron to determine the retained intron modulation. Additionally, another set of primers was designed in an exon region to determine total gene expression. The platform used in RT-qPCR assays was StepOnePlus (Life Technologies, Foster CA, USA), using the SYBR Green PCR Master Mix Kit (Life Technologies) and the reference genes *rpb2* and *gapdh*, as previously described [24]. The 2^−ΔΔCt^ relative quantification method [25] was used for the gene expression data, and graphs were generated using GraphPad Prism 5.

## 3. Results

### 3.1. In Silico Analyses

#### Characterization of the ABC Transporter Isoforms

**Figure 1:**
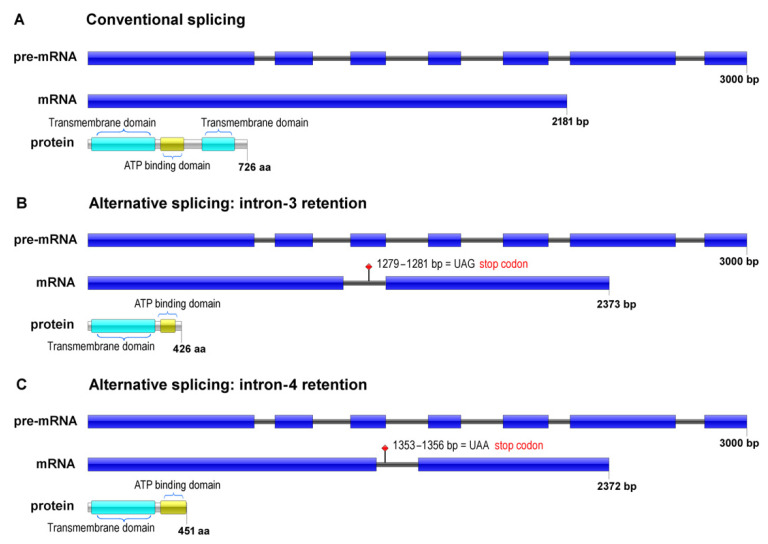
Schematic representation of Intron retention in TERG_04309 of *Trichophyton rubrum*, showing pre-mRNA, mRNA, and proteins resulting from conventional splicing (**A**) and alternative splicing promoted by Intron-3 retention (**B**) or intron-4 retention (**C**). Exons are shown as colored boxes; introns are shown as solid lines. Premature stop codons are indicated. Conserved sites and domains are indicated in the structure of the proteins.

RNA-seq data from *T. rubrum* exposed to the antifungal agents UDA and ACR suggested the occurrence of AS in genes encoding drug transporter proteins [3,15]. The gene encoding an efflux pump, identified as TERG_04309, was highlighted because it displays intron retentions with an expressive number of reads aligned in intron-3 and intron-4 regions [15]. This gene contained seven exons and six introns. The conventional spliced isoform was 2181 nucleotides in length, whereas the resulting alternative isoforms displayed 2373 and 2372 nucleotides for intron-3 and intron-4 retention, respectively (Figure 1).

TERG_04309 encodes an ABC-type drug transporter protein, characterized by the presence of a transmembrane domain and an ATP-binding domain. The protein resulting from conventional splicing has 726 amino acids, comprising two transmembrane domains (TMD) and an ATP-binding domain (NBD) (Figure 1A). The in silico characterization indicated that the retention of intron-3 and intron-4 produced smaller putative proteins containing 426 and 451 amino acids, respectively (Figure 1B,C). In our analysis, these proteins lost a transmembrane domain and part of the ATP-binding domain. 

**Figure 2:**
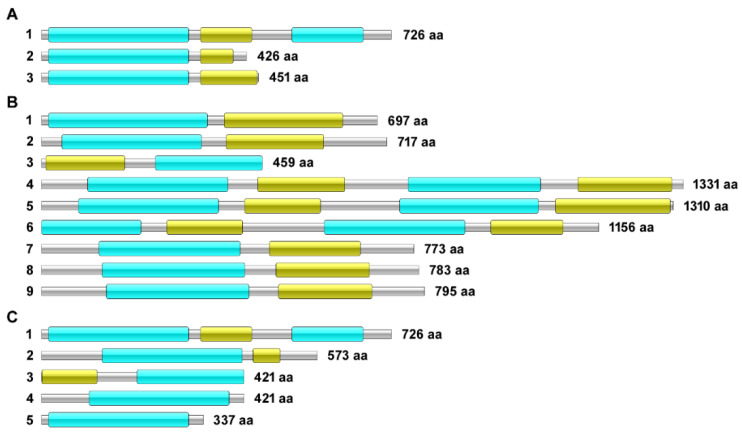
Protein isoforms of the TERG_04309 gene and their orthologs. (**A**) Protein isoforms of the TERG_04309 gene. (**A1**) Protein from conventionally processed isoform. (**A2**) Protein from alternative isoform (intron-3 retention). (**A3**) Protein from alternative isoform (intron-4 retention). (**B**) Orthologs of TERG_04309 protein in other species. **(B1**) *T. interdigitale* MR816 18 (H109_06718). (**B2**) *T. mentagrophytes* TIMM 2789 (TMEN_7207). (**B3**) *T. soudanense* CBS 452.61 (H105_04551). (**B4**) *Nannizzia gypsea* CBS 118893 (MGYG_04329). (**B5**) *Microsporum canis* CBS 113480 (MCYG_07490). (**B6**) *Neurospora crassa* str. 73. (GE21DRAFT_4932). (**B7**) *S. cerevisiae* (GCA_001634645) (WN66_06259). (**B8**) *C. albicans* 12C (MEK_04790). (**B9**) *C. auris* str. 6684 (QG37_04886). (**C**) The protein encoded by TERG_04309 in *T. rubrum* CBS118892 strain and orthologs in other *T. rubrum* strains. (**C1**) *T. rubrum* CBS118892 (TERG_04309). (**C2**) *T. rubrum* str. CMCC (F) T1i (A7C99_2094). (**C3**) *T. rubrum* CBS 735.88 (H106_04355). (**C4**) *T. rubrum* CBS 288.86 (H103_04538). (**C5**) *T. rubrum* CBS 202.88 (H107_04663). The transmembrane domains are represented in blue, and the ATP binding domains are indicated in green.

BLAST-P analysis comparing the protein encoded by TERG_04309 against orthologous sequences from dermatophytes and non-dermatophyte fungi is presented in Figure 2. These proteins show heterogeneity in their size and number of transmembrane and ATP-binding domains. *Nannizzia gypsea* (Figure 2B4), *Microsporum canis* (Figure 2B5), and *Neurospora crassa* (Figure 2B6) were shown to have the most extensive proteins among the species presented in this analysis, with four main domains each: two ATP-binding and two transmembrane domains. In contrast, the other six species contained proteins with only one transmembrane domain and one ATP-binding domain. Thus, most of the analyzed proteins showed this domain configuration and size.

Among *T. rubrum* species, the protein orthologs of TERG_04309 showed essential differences in structural organization, both for the protein extension and functional domains (Figure 2C). This protein has the highest molecular weight in *T. rubrum* CBS118892 compared with other *T. rubrum* strains. Such differences raise the possibility that these proteins act with different affinities or perform distinct functions that are yet to be revealed. 

In silico analysis using STRING software showed the interaction network of the TERG_04309 gene with ten other genes (Figure S1), including TERG_00588 that encodes a G-protein-coupled receptor (GPCR, protein-Ste2) and TERG_03468 (isoprenylcysteine O-methyltransferase—Ste14p). These interactions with TERG_04309 yielded scores of 0.636 and 0.319, respectively. Interestingly, another ABC transporter, encoded by TERG_04224, was identified using STRING software and showed a score of 0.280. These three genes were selected for further quantitative analysis (RT-qPCR).

### 3.2. Alternative Splicing in Response to Antifungals Exposure

**Figure 3:**
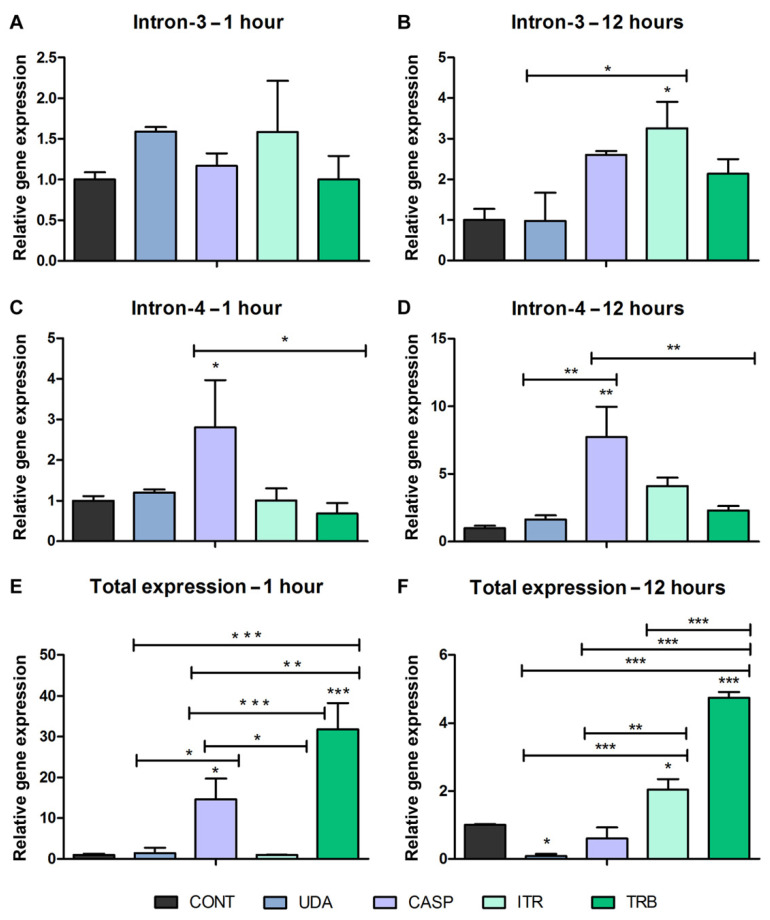
RT-qPCR analysis of isoform levels of the gene encoded by TERG_04309 in *T. rubrum*. Gene modulation data after 1 and 12 h of exposure to antifungal agents. (**A**,**B**) Expression levels of isoform with intron-3 retention. (**C**,**D**) Expression levels of the isoform with intron-4 retention. (**E**,**F**) Total expression levels of the TERG_04309 gene. Statistical analysis was performed using the *t*-test method. Asterisks refer to * *p* < 0.05, ** *p* < 0.01, and *** *p* < 0.001. As a reference for modulation, the condition of paired controls of 1 h and 12 h was used. Control sample (CONT); undecanoic acid (UDA); caspofungin (CASP); itraconazole (ITR), and terbinafine (TRB).

Quantitative analysis (RT-qPCR) of transcripts of the TERG_04309 gene revealed the induction of the isoform with retained intron-3 after *T. rubrum* exposure to ITR for 12 h (Figure 3B). Additionally, in response to CASP exposure, an increase in transcript levels of the isoform that retained intron-4 was observed at 1 and 12 h time points (Figure 3C,D). Transcript accumulation increased over time. This result demonstrates drug-dependent modulation of the isoforms. Furthermore, a notable increase in the transcript levels of the efflux pump TERG_04309 was observed in the conventional isoform after exposure of *T. rubrum* to TRB for 1 h and 12 h (Figure 3E,F). Transcript accumulation decreased over time. After 1 h of CAS and 12 h of ITR challenge, the efflux pump TERG_04309 induced transcript accumulation. In response to 12 h of UDA exposure, the transcripts of TERG_04309 were downregulated compared with those in the control (Figure 3E,F).

**Figure 4:**
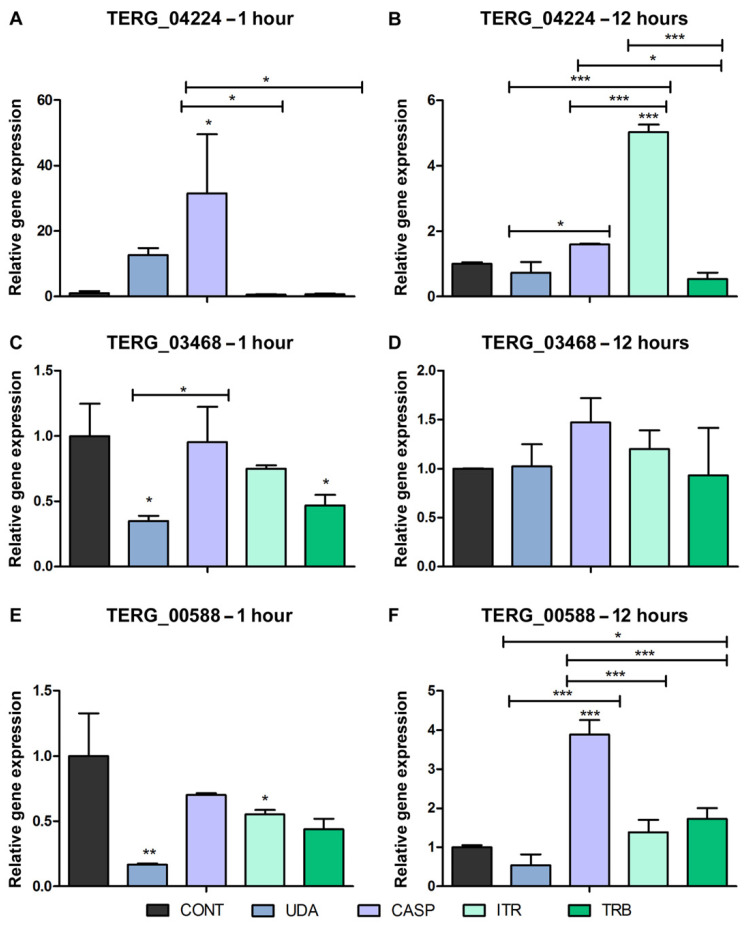
RT-qPCR analysis of expression levels of *T. rubrum* genes that putatively interact with the TERG_04309. Gene modulation data for TERG_04224 (**A**,**B**), TERG_03468 (**C**,**D**), and TERG_00588 (**E**,**F**) after 1 and 12 h of antifungal agent exposure. Statistical analysis was performed using the *t*-test method. Asterisks refer to * *p* < 0.05, ** *p* < 0.01, and *** *p* < 0.001. As a reference for modulation, the condition of paired controls of 1 h and 12 h was used. Control sample (CONT); undecanoic acid (UDA); caspofungin (CASP), itraconazole (ITR), and terbinafine (TRB).

Evaluation of the expression of the genes that putatively interact with the efflux pump TERG_04309 gene (Figure S1) showed that the ABC transporter TERG_04224 was induced in CASP and ITR after 1 h and 12 h of drug exposure, respectively (Figure 4A,B). This induction profile was also observed with retention of intron-4 of TERG_04309 after 1 h of CASP exposure (Figure 3C) and in the retained intron-3 after 12 h of ITR exposure (Figure 3B). These data validate the interaction between the two ABC transporters TERG_04309 and TERG_04224, previously observed in silico, and reveal that co-expression occurred with alternative isoforms of TERG_04309. 

The expression of two other genes that encode proteins that putatively interact with the ABC transporter TERG_04309, TERG_00588 (GPCR, protein-Ste2), and TERG_03468 (isoprenylcysteine O-methyltransferase—Ste14p) is also presented in Figure 4. TERG_03468 was downregulated after 1 h of UDA and TRB exposure (Figure 4C). No significant differences were observed after 12 h of exposure under any condition (Figure 4D). Our data indicate no interaction between TERG_04309 and TERG_03468. Transcript levels of TERG_00588 showed expressive induction after 12 h of CASP exposure (Figure 4F), similar to the retained intron-4 of TERG_04309 (Figure 3D). These data reiterate the co-expression of TERG_00588 with TERG_04309 identified using the STRING tool.

### 3.3. Alternative Splicing Evaluation in Response to Antifungal Exposure in the Biofilm Environment

**Figure 5:**
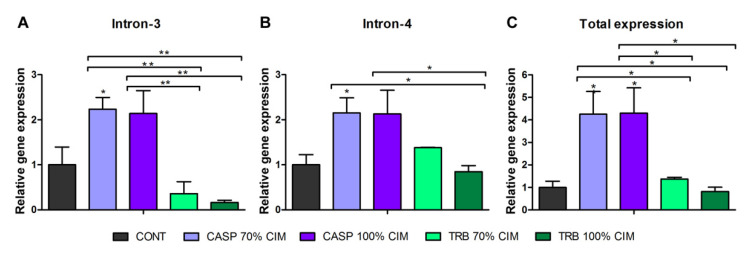
RT-qPCR analysis of the expression levels of different isoforms of the *T. rubrum* gene TERG_04309 after 1 h of biofilm exposure to antifungal agents. (**A**) Expression levels of isoform with intron-3 retention. (**B**) Expression levels of the isoform with intron-4 retention. (**C**) Total expression levels of the TERG_04309 gene. Statistical analysis was performed using the *t*-test method. Asterisks refer to * *p* < 0.05, and ** *p* < 0.01. As a reference for modulation, the condition of paired controls of 1 h and 12 h was used. The control sample (CONT); caspofungin (CASP), and terbinafine (TRB).

The TERG_04309 isoforms were also evaluated in the mature *T. rubrum* biofilm in response to CASP and TRB at concentrations corresponding to 70% of MIC and 100% of MIC. The use of MIC100 was because of the inherent characteristics of the biofilm being less susceptible to antifungal compounds [12,26]. CASP challenge induced the retention of both intron-3 and intron-4 (Figure 5A,B). Under the same conditions, increased expression of TERG_04309 was observed (total expression) (Figure 5C). Therefore, our results indicated the presence of three isoforms of TERG_04309 in response to CASP in the biofilm environment.

## 4. Discussion

Antifungal resistance involves several mechanisms, such as mutations in specific genes, upregulation of genes encoding efflux pumps, biofilm formation, and post-transcriptional modifications [2]. Alternative splicing is a post-transcriptional mechanism, and current studies have suggested its role in the stress adaptation caused by antifungal drugs in *T. rubrum* [3,16,18]. 

High-throughput data displayed the occurrence of AS in different *T. rubrum* cellular targets after UDA exposure, including in efflux pump transcripts [15]. Based on this data, we selected one gene that codes for an efflux pump, TERG_04309, to validate the occurrence of alternative splicing predicted in silico. This gene presented reads aligned to intron-3 at 12 h and to intron-4 after 3 and 12 h of UDA exposure, suggesting differential isoform modulation in response to the drug challenge.

The retention of intron-3 was observed exclusively in response to 12 h of ITR exposure. Conventional processing isoform transcript induction was equally observed in response to 12 h of ITR, suggesting a concurrent and time-responsive modulation to encounter the toxic effects of the drug. CASP exposure induced transcript accumulation of the isoform with retention of intron-4 at 1 and 12 h, with a more expressive transcript accumulation at 12 h. Differential expression of the conventional splicing isoform was observed exclusively in response to 1 h of CASP exposure. These results suggest that the traditional isoform of splicing actively induces CASP in a prompt response. In the latest experimental conditions, the alternatively spliced isoform (intron-4 retained) was apparently more active in extruding CASP. A significant accumulation of the conventional processing isoform was seen on TRB exposure at 1 h, with significantly decreased transcript accumulation at 12 h, revealing an inverted pattern of time-dependent accumulation of transcripts compared to intron-4 retention in response to CASP (Figure 3). UDA represses transcript accumulation of the conventional splicing isoform after 12-h of drug exposure, suggesting that other efflux pumps may be activated to counter the toxicity of this drug. These data revealed differences in isoform regulation according to compound structures related to differences in affinity or co-regulation with other multiple drug resistances (MDRs). It is well known that MDRs are modulated by their relationship with certain classes of antifungal agents [27].

The occurrence of intron retention has been previously demonstrated for other genes in *T. rubrum*, such as *pakA/Ste20*, *hsp7*, and *impdh* [15]. Intron retention in the mRNAs of these genes sometimes leads to a shift in the protein’s reading frame and premature stop codons, and consequently, the production of putative truncated proteins. Our analysis showed that the alternative isoforms of the TERG_04309 gene display premature stop codons and produce smaller proteins than the conventional isoform. ABC transporters have various topologies [7,28]. The topology of the protein isoforms encoded by TERG_04309 in *T. rubrum* is similar to some orthologous sequences from dermatophyte and non-dermatophyte fungi, suggesting that the structural organization of some ABC transporters might be influenced by AS events. Compensatory modulation of genes coding for ABC transporters in dermatophytes has been previously demonstrated. A mutant strain of *T. interdigitale* (Δ*mdr2*) enhanced transcription) of the *mdr4* gene after griseofulvin exposure, suggesting that the induction of *mdr4* would compensate for the inactivation of the *mdr2* gene [10]. Here, we identified that the ABC transporter gene (TERG_04224) expression was concomitantly induced with the following isoforms of TERG_04309: the retained intron 4 and the conventional isoform after 1 h of CASP challenge, and the retained intron 3 and the conventional isoform after 12 h of ITR exposure. These data revealed a synergistic activity of the ABC transporter TERG_04224 and the efflux pump TERG_04309 in *T. rubrum* in response to ITR and CASP. These data reinforce the complexity of drug transporter regulation in dermatophytes. Moreover, the ortholog of TERG_04309 in *T. equinum*, identified as TEQG_03450, was overexpressed when the fungus was exposed to ITR [10], suggesting its involvement in ITR efflux.

Fungi have sophisticated signaling cascades to sense and respond to different stresses, such as osmotic, temperature, UV irradiation, and oxidative stresses as well as exposure to antifungals [29]. The perception of stimuli is mainly mediated by GPCR signaling pathways. This is the largest class of cell surface receptors in fungi, consisting of receptor proteins characterized by TMD domains associated with intracellular G proteins [30]. Our results revealed that TERG_00588 interacts with the ABC transporter TERG_04309. TERG_00588 encodes a receptor protein (GPCR, Ste2) that is potentially involved in sensing cascades that regulate sexual reproduction pheromones in *S. cerevisiae* [31]. This receptor is also involved in chemotropism and virulence in *Fusarium graminearum* [32] and *Fusarium oxysporum* [33]. Herein, we showed the co-expression of TERG_00588 (Figure 4F) with the alternative isoform of TERG_04309, which retained intron-4 in response to 12 h of CASP exposure (Figure 3D), suggesting interactions between these genes. These data raise the possibility that Ste2 plays a role in antifungal sensing.

The modulation of the conventional, as well as different isoforms of the TERG_04309 gene in mature *T. rubrum* biofilms exposed to CASP (Figure 5), suggests a net and highly connected signaling in these structures and adaptation to antifungals. This modulatory pattern may be produced in biofilms to counteract CASP stress. We hypothesized that the simultaneous production of these isoforms might be associated with the abundance of glucan in the biofilm matrix. This triggers fine regulation through AS in ABC transporter genes to cope with an antifungal that acts by blocking the synthesis of (1,3)- β-d-glucan in the fungal cell wall.

Other studies have highlighted the biofilm-associated antifungal resistance, as shown in *M. canis*, *T. mentagrophytes* [12], *T. rubrum* [34], and *C. auris* [26]. The mechanisms associated with antifungal resistance are the physical barrier conferred by the glucan matrix as well as cell signaling, which enhances regulatory mechanisms in this population [13]. Herein, we shed light on another level of regulation promoted by biofilm structures that evolve into AS events. The occurrence of AS in this efflux pump-encoding gene and the synergistic production of its isoforms in fungal biofilms suggest the high complexity of occurrence of drug tolerance. It expands our understanding of the regulation of efflux pumps. Our findings reveal in *T. rubrum* a new mechanism of resistance or adaptation to antifungals. However, other species of fungi must be analyzed to verify the amplitude of this phenomenon. 

**Figure 6:**
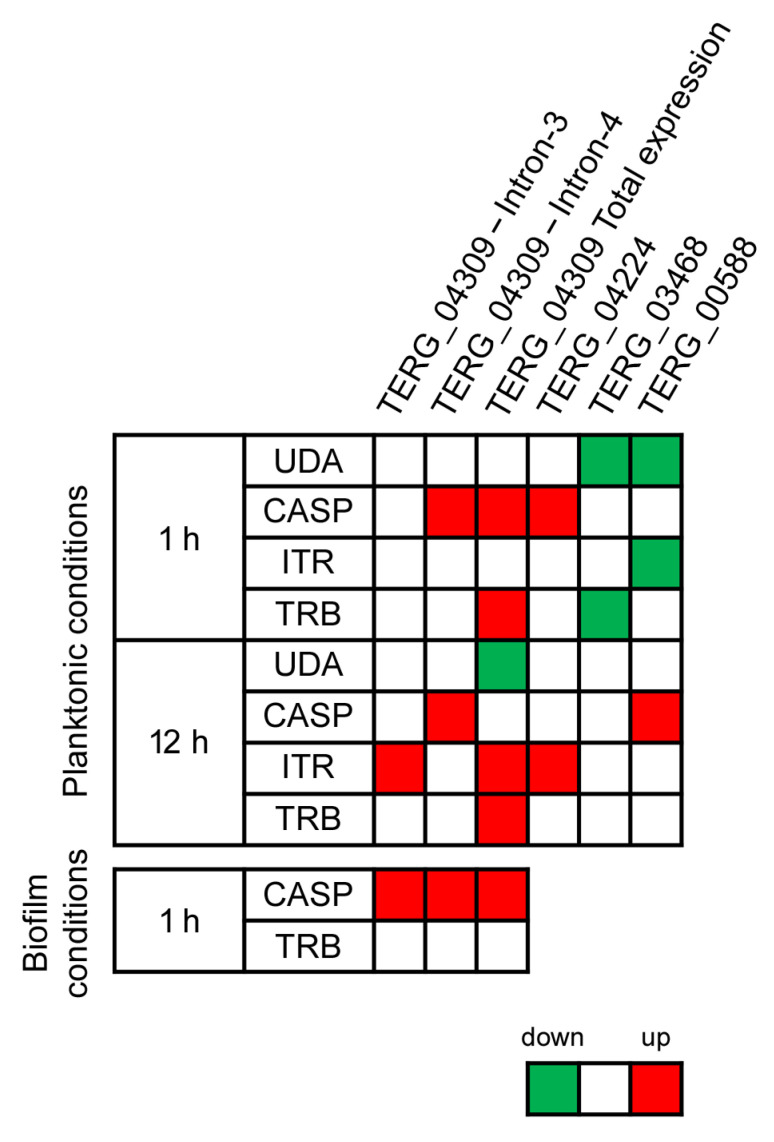
Summary of the main results of gene expression in response to antifungals challenge.

Figure 6 summarizes the gene expression data obtained in the planktonic and biofilm conditions.

## 5. Conclusions

Our data showed that intron retention acts as a regulatory mechanism for drug transporters. The simultaneous production of isoforms in response to drug exposure leads to a synergistic effect that evolves adaptive responses to cope with the stress caused by antifungals. AS can ensure quick and effective responses to different stress conditions by producing a vast repertoire of isoforms, which may be associated with greater antifungal tolerance, mainly in biofilms. Further investigations should be conducted to unveil the signaling and activated circuits that govern and trigger isoform production.

## Data Availability

The data presented in this study are available in this article and the accompanying Supplementary Data.

## Supplementary Materials

The following are available online at https://www.mdpi.com/article/10.3390/jof8080878/s1, Table S1. Primers used in RT-qPCR assays. Figure S1. Interaction network of the *T. rubrum* TERG_04309 gene (Transporter ABC) with other genes. Next to each gene, the interaction score obtained is indicated (in bold). The colors of the interaction lines refer to the putative forms of that interaction (STRING software).
