# Supplementary material for: Alternative Splicing in Trichophyton rubrum Occurs in Efflux Pump Transcripts in Response to Antifungal Drugs
Source: J Fungi (Basel). 2022 Aug 20;8(8):878. doi: 10.3390/jof8080878 (PMC9410333; doi:10.3390/jof8080878)
Supplement: Supplementary file 1 [file jof-08-00878-s001.zip › jof-1829436-supplementary.pdf]

# Alternative splicing occurs in the efflux pump transcripts in response to antifungal drugs

## Supplementary Materials

**Table S1.** Primers used in RT-qPCR assays.

| ID | Primers | Amplicon | Concentration (nM) | Reference |
|---|---|---|---|---|
| TERG_04309 (ABC transporter)<br>Total expression | F: GGGTATCGTGTTGAAGCTAAGA<br>R: CATCCAGAAAGACAGGCCATAA | 114 bp | 100 nm | This work |
| TERG_04309 (ABC transporter)<br>(Intron-3) | F: ACACGGATTAATCGGAACACC<br>R: AGCACAGGTATACTCACGTTTG | 101 bp | 100 nm | This work |
| TERG_04309 (ABC transporter)<br>(Intron-4) | F: ACTCGATTAAAGAGCGAAGGA<br>R: CCGCATTCTTGATAGGAGAGA | 106 bp | 300 nm | This work |
| TERG_04224 (ABC transporter)<br>Total expression | F: CAACTGCTCTCCGTTCTT<br>R: CTTCCGTCGATAGAGAATCG | 125 bp | 100 nm | This work |
| TERG_03468 (Ste14p O-methyltransferase)<br>Total expression | F: CAACTGCTCTCCGTTCTT<br>R: CTTCCGTCGATAGAGAATCG | 100 bp | 400 nm | This work |
| TERG_00588 (Ste2 – Pheromone Receptor)<br>Total expression | F: TCGAGCTATCCCTACTCATC<br>R: AATTGGAACCGCTGCTAC | 107 bp | 100 nm | This work |
| TERG_04402 (*gapdh*) | F: GCGTGACCCAGCGATGTAGT<br>R: CGGTGGACTCGACGATGTAGT | 62 bp | 200 nm | [24] |
| TERG_05742 (*rpb2*) | F: TGCAGGAGGTTTGATGAAGA<br>R: GCTGGGAGGTACTGTTTGATCAA | 59 bp | 300 nm | [24] |

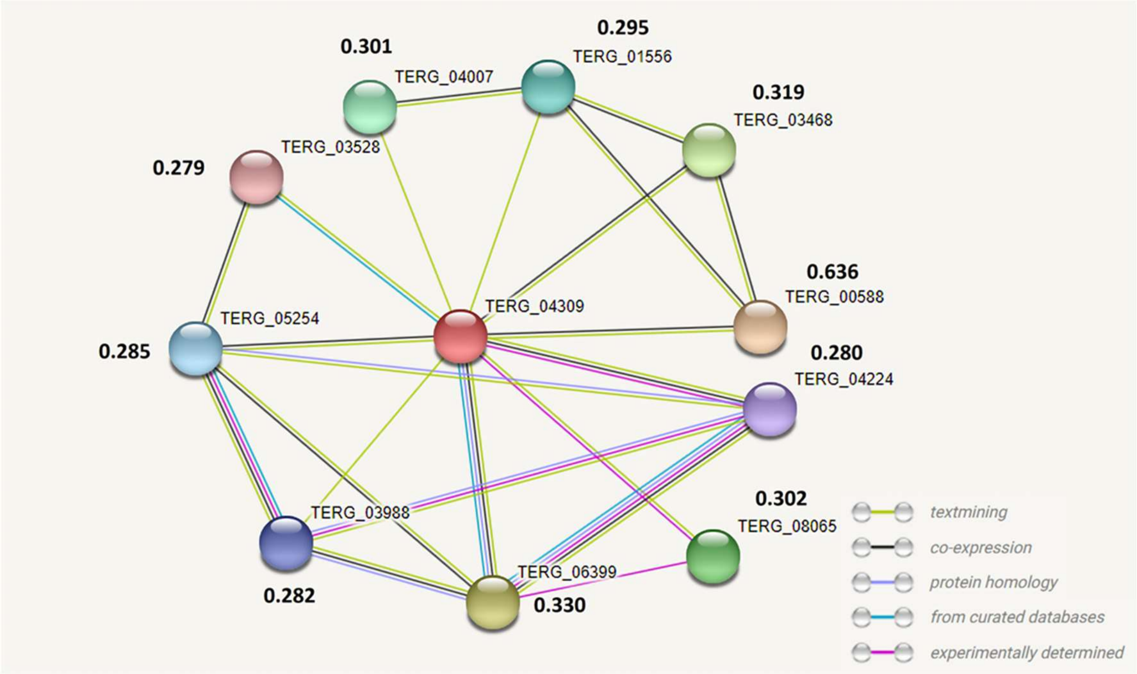


**Figure S1.** Interaction network of the *T. rubrum* TERG_04309 gene (Transporter ABC) with other genes. Next to each gene, the interaction score obtained is indicated (in bold). The colors of the interaction lines refer to the putative forms of that interaction (STRING software).
